# Pleural involvements in pulmonary sarcoidosis: A case report and review of the literature

**DOI:** 10.3389/fmed.2022.902711

**Published:** 2022-11-16

**Authors:** Xiaoqing Ji, Jiameng Lu, Anli Zuo, Fei Sun, Haiying Peng, Degan Lu

**Affiliations:** ^1^Department of Nursing, The First Affiliated Hospital of Shandong First Medical University and Shandong Provincial Qianfoshan Hospital, Jinan, China; ^2^School of Microelectronics, Shandong University, Jinan, China; ^3^Department of Respiratory, The First Affiliated Hospital of Shandong First Medical University and Shandong Provincial Qianfoshan Hospital, Shandong Institute of Respiratory Diseases, Jinan, China; ^4^Department of Pulmonary and Critical Care Medicine, Jining First People’s Hospital, Jining, China; ^5^Department of Respiratory Medicine, The Second People’s Hospital of Yibin, Yibin, China; ^6^Department of Respiratory, The First Affiliated Hospital of Shandong First Medical University and Shandong Provincial Qianfoshan Hospital, Shandong Institute of Respiratory Diseases, Shandong Institute of Anesthesia and Respiratory Critical Medicine, Jinan, China; ^7^Department of Gerontology, The First Affiliated Hospital of Shandong First Medical University and Shandong Provincial Qianfoshan Hospital, Shandong Institute of Respiratory Diseases, Shandong Institute of Anesthesia and Respiratory Critical Medicine, Jinan, China

**Keywords:** sarcoidosis, pleural effusion, pleural nodules, biopsy, thoracoscopy

## Abstract

As a chronic and multisystemic granulomatosis of unknown origin, sarcoidosis can affect multiple organs throughout the body with variable progression and prognosis. Sarcoidosis may present with a battery of symptoms and signs, such as dyspnea, non-productive cough, uveitis, and erythema nodosum. Although the lungs and mediastinal lymph nodes are almost affected in sarcoidosis, involvements of the pleurae remain uncommon. Herein, we report a case of sarcoidosis with both pleural effusions and pleural nodules as confirmed by thoracoscopic pleural biopsy.

## Introduction

Sarcoidosis is a multisystem disease characterized by non-caseating granulomatous inflammation and its exact etiology remains unknown (1). It can affect different organs, especially the lungs, lymph nodes, skin, and eyes. Although lungs are involved in approximately 90% of patients with sarcoidosis, the pleurae are rarely affected in this perplexing disease (2). The frequency of pleural sarcoidosis was less than 3% and pleural effusion, pleural thickening, pleural nodules, and pneumothorax were major patterns of pleural involvements (3, 4). Sarcoidosis presenting as both pleural effusion and pleural nodules are even more unusual. The present report describes a case of sarcoidosis with pleural nodules and pleural effusion.

## Case report

**FIGURE 1:**
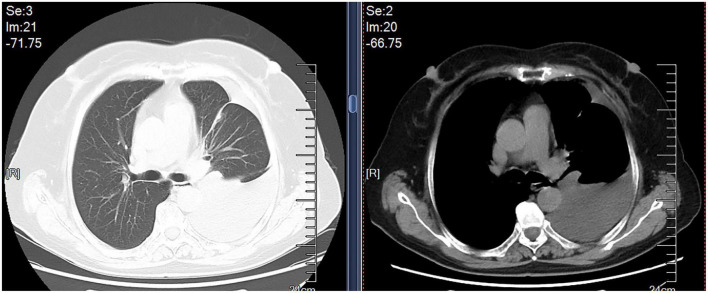
Chest CT scan showed left pleural effusions and linear opacity.

**FIGURE 2:**
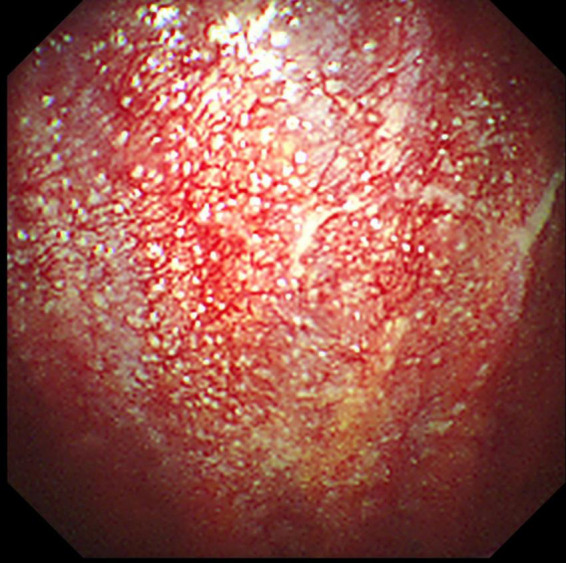
Thoracoscopy demonstrated amounts of white nodules on the pleurae.

**FIGURE 3:**
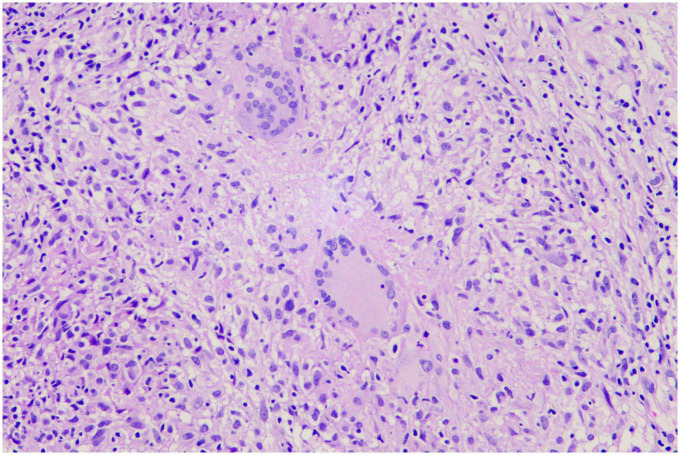
Histologic finding of non-caseating granulomas (hematoxylin-eosin, original magnification × 400).

**FIGURE 4:**
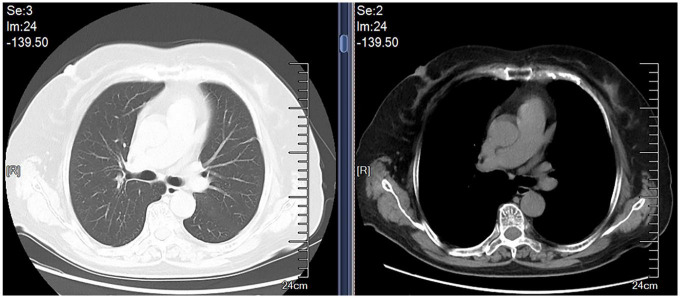
Follow-up chest CT showed a complete withdrawal of the left pleural effusion.

A 69-year-old female was admitted to our hospital in Oct 9, 2019 because of dry cough, dyspnea on exertion and fatigue for a month. She had no night sweats or weight loss. The patient was a nurse and she had neither previous disease nor history of smoking. She also had no history of exposure to foreign antigens inorganic particulates. In addition, she had no family history of rheumatologic or autoimmune diseases. On admission, temperature, pulse, respiratory rate, and blood pressure were 36.6°C, 79 beats/min, 21 breaths/min, and 126/88 mm Hg, respectively. Serum levels of electrolytes, brain natriuretic peptide, and liver & kidney function were normal. The levels of serum carcino-embryonic antigen (CEA), neuro-specific enolase (NSE), CYFRA 21-1, squamous cell carcinoma antigen (SCCA), and pro-gastrin-releasing peptide (Pro-GRP) were 1.24 ng/ml (0–5.0 ng/ml), 17.78 ng/ml (0–16.3 ng/ml), 1.54 ng/ml (0.1–3.3 ng/ml), 0.9 ng/ml (0–1.5 ng/ml), and 34.19 pg/ml (0–63 pg/ml), respectively. A computed tomographic (CT) scan of the thorax demonstrated left pleural effusions and linear opacity (Figure 1). The exudative, yellow fluid with the predominance of lymphocytes (85%) was detected by thoracentesis. The concentrations of CEA, NSE, CYFRA 21-1, SCCA, and Pro-GRP in pleural effusion were 0.62 ng/ml (0–5.0 ng/ml), 0.86 ng/ml (0–16.3 ng/ml), 11.95 ng/ml (0.1–3.3 ng/ml), 2.5 ng/ml (0–1.5 ng/ml), and 34.72 pg/ml (0–63 pg/ml), respectively. The levels of total protein, lactic dehydrogenase (LDH), glucose, and adenosine deaminase (ADA) in pleural effusion were 54.1 g/L (serum level in the same day was 66.80 g/L), 208 units/L (serum level in the same day was 200.0 units/L), 6.96 mmol/L (3.6–6.0 mmol/L), and value was 47.5 IU/L (<45 IU/L). PR3-anti-neutrophil cytoplasmic antibodies (ANCA) and MPO-ANCA were both negative in the serum. Cultures of the pleural effusion were negative for acid fast bacilli (AFB), fungi, and other organisms. A purified protein derivative (PPD) skin test was also negative. The cytological examination of exfoliated cells in pleural fluid revealed no malignant cells. To clarify the cause of pleural effusion, video-assisted thoracoscopy was performed and revealed a myriad of white nodules on both the visceral and parietal pleura, as well as on the diaphragm (Figure 2). Histological examination of the pleural biopsy sample demonstrated non-caseating granulomas and real-time quantitative polymerase chain reaction (PCR) for mycobacterium tuberculosis was negative (Figure 3). Stains for acid fast bacilli and fungi remained negative. These results, together with no evidence of other granulomatous disorders, were believed to be consistent with pleural sarcoidosis and this patient was started on prednisone 30 mg daily. All complaints regressed 2 weeks later and a follow-up CT 4 weeks later exhibited the left pleural effusion almost disappeared (Figure 4).

## Discussion

The clinical features of pleural involvements in our patient seem to be interesting for several reasons. Firstly, this case of sarcoidosis is presented with both pleural effusion and pleural nodules. Pleural involvement in sarcoidosis was first recognized by Schaumann in 1933 and it can be manifested by pleural effusion, pleural nodules, pleural thickening, pneumothorax, hydropneumothorax, and chylothorax (4, 5). The true incidence of pleural involvements in sarcoidosis remains unclear because some cases of pleural sarcoidosis are asymptomatic (6). Chusid and Siltzbach reported that pleural involvements were histologically confirmed in 5 of 950 (0.5%) patients with sarcoidosis (7). Soskel et al. stated that pleural involvement occurred in about 3% of sarcoidosis). The first case of histologically proven pleural effusion caused by sarcoidosis was reported by Talbot et al. (8). Sarcoidosis-related pleural effusions were considered to be less than 3% of this entity and, when present, occur slightly more commonly in the right pleural cavity, although sometimes they can be bilateral (4). The typical finding in pleural effusions caused by sarcoidosis is a paucicellular exudate with the predominance of lymphocytes (9). The mechanism of pleural effusion formation may be analogous to that of other infiltrative diseases. Increased capillary permeability due to involvement of the pleura, obstruction of superior vena cava, lobar atelectasis, and trapped lung have been considered as a cause of pleural effusions secondary to sarcoidosis (10, 11). Pleural nodules, another manifestation of pleural sarcoidosis, were infrequent although the use of CT and thoracoscopy has increased awareness of this unusual site of involvement in sarcoidosis. These are often described as innumerable white nodules on both the parietal and visceral pleura (12, 13). Sarcoidosis-related pleural effusions and pleural nodules are unusual, and they occur concurrently in one patient, as described in our case, are even more uncommon.

Secondly, pleural involvement is the initial manifestation of sarcoidosis. Pleural manifestations caused by sarcoidosis may arise at the onset of this disease which is first diagnosed, as the case we have described, or at any time during the course of the known sarcoidosis (14). The development of pleural involvement in sarcoidosis seems to have no definite prognostic value (15). Thirdly, the pleural effusion and pleural nodules are associated with neither hilar adenopathy nor pulmonary infiltrate. The most common radiographic finding of sarcoidosis is bilateral hilar adenopathy. Other clinical features consist of interstitial lung disease, pulmonary nodules, skin lesions, and eye symptoms (12). Pleural sarcoidosis usually correlates with extrapulmonary involvement or extensively parenchymal lesions of the lung (7, 9, 16). In the present case, of great interest is pleural involvements are not associated with hilar adenopathy or pulmonary infiltrate.

Finally, the pleural involvement of sarcoidosis responds well to corticosteroids. Systemic corticosteroids are the mainstay of treatment of sarcoidosis and also the most commonly used first-line therapy (17). Corticosteroids hamper the formation of granulomas and, as a result, are largely efficient against most active clinical manifestations (18). For recurrent or symptomatic patients of sarcoidosis with pleural involvement, corticosteroids are required (19). Asymptomatic pleural effusions are likely to resolve spontaneously. The time of spontaneous resolution ranges from 1 to 3 months (19). Our case responded well to oral corticosteroid therapy, resulting in marked improvement in both symptoms and chest radiological findings. Sarcoidosis related pleural effusions which resolve incompletely and develop to trapped lung may be relieved by decortication (11).

The diagnosis of sarcoidosis involving the pleura is based on the histologic evidence of non-caseating granuloma, the hallmark of sarcoidosis, and on excluding other granulomatous diseases, such as tuberculosis, fungal disease, and granulomatous polyvasculitis (2). In addition, some disorders including congestive heart failure and neoplasia, may be concomitant with sarcoidosis, must be ruled out. Our case showed no evidences of tuberculosis, fungal disease, vasculitis, and other granulomatous diseases. However, because the clinical and pathological features of sarcoidosis and tuberculosis may mimic each other, the differentiation between the two entities remains a challenging problem. When the caseous necrosis is absent in biopsy samples, the real time PCR quantification for mycobacterium tuberculosis genome is a valuable test for differentiation between sarcoidosis and tuberculosis (20).

Medical thoracoscopy, a relatively less invasive and more efficient diagnostic method, plays a significant role in the diagnosis of pleural involvement in sarcoidosis, especially of pleural effusions and pneumothorax. By thoracoscopy, physicians can directly access and assess the pleural cavity, including the parietal, visceral and diaphragmatic pleura, and obtain adequate tissue sampling. Additionally, pleural fluid can be aspirated without complications during thoracoscopy. Therefore, thoracoscopy, an appropriate alternative technique, can provide doctors with important evidences to convince pleural sarcoidosis (21). Although thoracentesis or closed pleural biopsy can also help to diagnose, it is not easy for physicians to get the accurate pathologic evidence.

## Conclusion

In summary, this case illustrates an unusual form of pleural involvement of sarcoidosis with pleural effusion plus pleural nodules. A definitive diagnosis of pleural sarcoidosis relies on the histological identification of non-caseating granulomas in the pleurae and on the exclusion of all other possible causes. Although rare, pleural involvements in sarcoidosis should be considered in the differential diagnosis of pleural effusion and pleural nodules. As is the case with other forms of pulmonary involvement in sarcoidosis, these manifestations respond well to corticosteroids. It is believed that careful evaluation and vigorous treatment of pleural involvement in sarcoidosis is imperative.

## Data availability statement

The raw data supporting the conclusions of this article will be made available by the authors, without undue reservation.

## Ethics statement

The studies involving human participants were reviewed and approved by the First Affiliated Hospital of Shandong First Medical University (Jinan, China). The patients/participants provided their written informed consent to participate in this study. Written informed consent was obtained from the individual(s) for the publication of any potentially identifiable images or data included in this article.

## Author contributions

XJ, JL, and DL: conception and design. XJ, JL, HP, and FS: collection and assembly of data. XJ, JL, AZ, and DL: data analysis and interpretation. All authors: manuscript writing and final approval of manuscript.
